# Genomic-Enabled Prediction Kernel Models with Random Intercepts for Multi-environment Trials

**DOI:** 10.1534/g3.117.300454

**Published:** 2018-02-23

**Authors:** Jaime Cuevas, Italo Granato, Roberto Fritsche-Neto, Osval A. Montesinos-Lopez, Juan Burgueño, Massaine Bandeira e Sousa, José Crossa

**Affiliations:** 1 Universidad de Quintana Roo, Chetumal, Quintana Roo, México; 2 Department of Genetics, Luiz de Queiroz College of Agriculture, University of São Paulo, Piracicaba, São Paulo, Brazil; 3 Facultad de Telemática, Universidad de Colima, CP 28040 Colima, Edo. de Colima, México; 4 Biometrics and Statistics Unit, International Maize and Wheat Improvement Center (CIMMYT). Apdo. Postal 6-641, 06600 México DF, México

**Keywords:** Genomic-enabled prediction accuracy, genotype × environment interaction, main genetic effects, deviations from main genetic effects, random intercepts, Genomic Selection, shared data resource, GenPred

## Abstract

In this study, we compared the prediction accuracy of the main genotypic effect model (MM) without G×E interactions, the multi-environment single variance G×E deviation model (MDs), and the multi-environment environment-specific variance G×E deviation model (MDe) where the random genetic effects of the lines are modeled with the markers (or pedigree). With the objective of further modeling the genetic residual of the lines, we incorporated the random intercepts of the lines (l) and generated another three models. Each of these 6 models were fitted with a linear kernel method (Genomic Best Linear Unbiased Predictor, GB) and a Gaussian Kernel (GK) method. We compared these 12 model-method combinations with another two multi-environment G×E interactions models with unstructured variance-covariances (MUC) using GB and GK kernels (4 model-method). Thus, we compared the genomic-enabled prediction accuracy of a total of 16 model-method combinations on two maize data sets with positive phenotypic correlations among environments, and on two wheat data sets with complex G×E that includes some negative and close to zero phenotypic correlations among environments. The two models (MDs and MDE with the random intercept of the lines and the GK method) were computationally efficient and gave high prediction accuracy in the two maize data sets. Regarding the more complex G×E wheat data sets, the prediction accuracy of the model-method combination with G×E, MDs and MDe, including the random intercepts of the lines with GK method had important savings in computing time as compared with the G×E interaction multi-environment models with unstructured variance-covariances but with lower genomic prediction accuracy.

Genomic selection (GS) predicts breeding values of complex traits based on dense marker information (Meuwissen *et al.* 2001) and has shown good prediction accuracy achieved by random cross-validation partitions of plant breeding data (de los Campos *et al.* 2009, 2013; Crossa *et al.* 2010, 2011; 2013; Pérez-Rodríguez *et al.* 2012). As molecular markers become cheaper and more abundant, GS-assisted breeding has become commonly used in plant and animal improvement. When performing genomic prediction of breeding values of unobserved individuals, the relationship between individuals in the training and testing sets is computed through the genomic relationship matrix, and the prediction model is referred to as the Genomic Best Linear Unbiased Predictor (GBLUP) (VanRaden, 2007, 2008).

Multi-environment trials are routinely conducted in plant breeding to estimate and take advantage of genotype × environment interaction (G×E) for selecting stable and high performing lines across environments and within environments. Therefore, implementation of GS strategies in plant breeding should be useful for estimating the parameters of the model and predicting G×E, as is commonly done in conventional plant breeding. Modern statistical analyses of multi-environment trials assess G×E by using pedigree information with linear mixed models (Piepho, 1997, 1998; Smith *et al.* 2005; Crossa *et al.* 2006; Burgueño *et al.* 2007); however, these models do not incorporate marker information.

A Bayesian GBLUP regression model for assessing genomic-enabled prediction combining G×E introduces the main effects of environments and lines and the interaction effects of markers and environmental co-variables via random variance-covariance structures (Jarquín *et al.* 2014). The Bayesian regression model of López-Cruz *et al.* (2015) is similar to that of Jarquín *et al.* (2014) with one difference: that genomic values are partitioned into components that are stable across environments (main genomic effects) and others that are environment-specific (genomic G×E) (Crossa *et al.* 2016). Although both models assume positive sample correlations among environments and can be fitted using the BGLR package (de los Campos and Pérez-Rodríguez 2016), the advantage of the model of López-Cruz *et al.* (2015) over the model of Jarquín *et al.* (2014) is that it can be implemented using both shrinkage methods and variable selection methods and is efficient when applied to sets of environments that have positive correlations because the genetic covariance between any pair of environments is the variance of the main effect, which makes the covariance between pairs of environments positive (López-Cruz *et al.* 2015).

Cuevas *et al.* (2016) used the Bayesian model of López-Cruz *et al.* (2015) to compare methods that apply GS models with G×E using a linear kernel (GBLUP) (GB) and a non-linear Gaussian kernel (GK) for single-environment and multi-environment breeding data sets. The authors found the GK models had higher prediction accuracy than the GB models and explained that the GK models captured major and complex marker effects in addition to their interaction effects. Sousa *et al.* (2017) compared the prediction accuracy of the multi-environment, single variance G×E deviation model (MDs) of Jarquín *et al.* (2014) with GK (MDs-GK) and the prediction accuracy of the multi-environment environment-specific variance G×E deviation model (MDe) of López-Cruz *et al.* (2015) with the GK method (MDe-GK). Then, Sousa *et al.* (2017) compared the models including the GK method with the prediction accuracy of their counterpart models using the GB methods (MDs-GB and MDe-GB). In addition, Sousa *et al.* (2017) also compared the accuracy of the four previous models with the accuracy of the multi-environment, main genotypic effect (MM) of Jarquín *et al.* (2014) using the GB and GK methods (MM-GB, and MM-GK). Results show that for grain yield, a notable increase in prediction accuracy of GK over the GB methods ranged from 9 to 49% in one data set and from 34 to 70% in another data set.

In general, the previous linear mixed multi-environment models assumed the environments as fixed or random effects, and lines as random effects incorporating into the model the random slope of the genetic effect of the lines distributed as a normal random variable with zero mean and variance-covariance structure constructed from markers or pedigree; also, the genetic effect (intercept) of the lines can be considered as having a normal distribution with zero mean and constant variance (Mota *et al.* 2016). The random intercept of the lines is often not included in the model when no exchange of information occurs, assuming the intercepts are independent (Pérez-Rodríguez *et al.* 2015). However, recent studies have incorporated random intercepts (Mota *et al.* 2016; Cuevas *et al.* 2017; Sukumaran *et al.* 2017; Jarquín *et al.* 2017) in order to achieve higher genomic-prediction accuracy in cases where lines were observed in some environments but not in others (random cross-validation 2, CV2 of Burgueño *et al.* 2012); this is because the posterior distribution of the intercept generates a variance-covariance structure that allows exchanging information between the lines of the training and testing sets. When newly developed lines have never been observed (untested) (random cross-validation CV1, Burgueño *et al.* 2012), models do not improve the prediction accuracy with or without random intercept when compared with the single-environment model. One limitation of these multi-environment genomic G×E models for achieving relatively high genomic-enabled predictions is that correlations among environments should be positive. Also, none of the applications of the models of Jarquín *et al.* (2014), Sukumaran *et al.* (2017), and Jarquín *et al.* (2017) compared genomic-enabled prediction accuracy with GB kernel *vs.* GK kernel.

The previous Bayesian regression models of Jarquín *et al.* (2014) and López-Cruz *et al.* (2015) use the Hadamard product for modeling G×E and show that the exchange of information between environments is achieved by means of the variance-covariance matrix of the main effects. Thus, the variance component of the main effects measures the stability across environments and the variance component of the specific effects measures the deviations from the main effects due to specific combinations of lines in environments (G×E). This approach has the advantage that it can be used when the number of lines in each environment is the same, but also when there is an unbalanced number of lines in environments, as shown by Sousa *et al.* (2017).

On the other hand, GBLUP methodology (together with pedigree) can incorporate and model G×E effects, by means of the Kronecker product of the variance-covariance matrices of the genetic relationship between environments and the genomic or pedigree relationship between the lines (Burgueño *et al.* 2012; Oakey *et al.* 2016) where the structure of the models allows estimating negative genetic correlations between environments. Based on this, Cuevas *et al.* (2017) recently compared a Bayesian regression model for the genetic effects described by the Kronecker product of unstructured variance-covariance matrices of genetic correlations between environments and genomic kernels under the GB and GK methods. An extension includes an extra genetic residual component with random intercepts. Results of the analyses of five data sets indicated that including the random intercepts is still beneficial for increasing genomic prediction accuracy when lines have been tested in some environments. However, one drawback of the Bayesian regression models of Cuevas *et al.* (2017) is the computing time for the iteration required for the Monte Carlo Markov Chain (MCMC) method to achieve the convergence of the posterior and predictive distributions.

Recently Granato *et al.* (2017) proposed an R package called Bayesian Genomic G×E (BGGE) to obtain a rapid fit of Bayesian mixed linear models with homogeneous error variances for the models of Jarquín *et al.* (2014), López-Cruz *et al.* (2015) and also for the models used by Sousa *et al.* (2017) (MM, MDs, and MDE). The approach of Granato *et al.* (2017) uses an R library that saves time by using the structure of the block diagonal matrices with additional parameterizations to shorten the iteration time without losing precision.

Based on the above, the main objective of this study was to compute the prediction accuracy of 16 model-method combinations and compare their prediction accuracy for four different data sets (two maize and two wheat multi-environment trials) with an unbalanced number of lines in environments, and different complexity of the G×E interaction. The 16 model-methods comprise the multi-environment, main genotypic effect (MM), the multi-environment, single variance G×E deviation model (MDs) and the multi-environment environment-specific variance G×E deviation model (MDe) with the GB and GK kernel methods and with and without including random intercepts (12 model-methods) plus 4 Bayesian regression models for the genetic effects described by the Kronecker product of unstructured variance-covariance (MUC) matrices of genetic correlations between environments and genomic kernels under the GB and GK methods and their extensions, including an extra genetic residual component with random intercepts. We discuss the advantages and disadvantages of the different model-methods for sets of environments with different G×E characteristics and different degrees of unbalance among lines.

## Materials and Methods

This study uses four multi-environment plant breeding data sets with different characteristics. Two maize data sets used by Sousa *et al.* (2017) (HEL and USP) had different numbers of maize hybrids in each environment and positive correlations between environments, whereas the two wheat data sets used by Cuevas *et al.* (2017) (WHE1 and WHE5) had environments with negative or zero correlations but with the same number of wheat lines in each location.

We used the same models of Sousa *et al.* (2017) (MM, MDs, and MDe) with linear (GB) and non-linear kernels (GK) (MM-GB, MM-GK, MDs-GB, MDs-GK, MDe-GB, MDe-GK) plus the addition of one random intercept component (l) that captures the variation of genetic residuals (MMl-GB, MMl**-**GK, MDsl-GB, MDsl-GK, MDel-GB, MDel-GK). These 12 model-methods were fitted with the BGGE package (Granato *et al.* 2017).

In this study models 2 and 3 of Cuevas *et al.* (2017) are renamed as Multi-environment Unstructured Covariance (MUC) and Multi-environment Unstructured Covariance with random intercept vector ***f*** (MUC***f***), respectively, each fitted with the GB and GK kernel methods. Therefore, 4 additional models are included, MUC-GB, MUC-GK, MUC***f***-GB, and MUC***f***-GK. These models were fitted with the MTM package (de los Campos and Grüneberg 2016) and their prediction accuracy was compared with the other 12 model-method combinations.

In the first step, the phenotypic data were fitted according to the experimental design employed for each experiment, and the Best Linear Unbiased Estimates (BLUE) of the lines or hybrids for each location or environments were computed. In the second step, the various genomic models were fitted to perform random cross-validation and compute the prediction accuracy of the 16 model-method combinations.

### Experimental data

#### Maize data set HEL:

This maize data set comprises 452 maize hybrids evaluated in 2015 at five sites in Brazil: Nova Mutum (NM) and Sorriso (SO) in the state of Mato Grosso; Pato de Minas (PM) and Ipiaçú (IP) in the state of Minas Gerais; and Sertanópolis (SE) in the state of Paraná. The experimental design was a randomized block with two replicates per genotype and environment. Different numbers of hybrids were planted in each environment. The HEL parent lines were genotyped with an Affymetrix Axiom Maize Genotyping Array of 616 K SNPs with standard quality controls removing markers with a *Call Rate ≥* 0.95.

#### Maize data set USP:

This data set comprises 740 maize hybrids evaluated at Piracicaba and Anhumas, each with two levels of nitrogen (N) fertilization: Ideal N (IN) and Low N (LN) for a total of four artificial environments (P-IN, P-LN, A-IN, and A-IN). The hybrids were evaluated using an augmented block design including two replicated commercial hybrids as checks. There was an imbalance because not all hybrids were evaluated in all locations. Similar to the maize data set HEL, the USP parent lines were genotyped with an Affymetrix Axiom Maize Genotyping Array of 616 K SNPs with standard quality controls removing markers with a *Call Rate ≥* 0.95.

#### Wheat data set WHE1:

A historical set of 599 wheat lines from CIMMYT’s Global Wheat Program was evaluated in four mega-environments (Crossa *et al.* 2010; Cuevas *et al.* 2016) and genotyped using 1447 Diversity Array Technology (DArT) markers generated by Triticarte Pty. Ltd. (Canberra, Australia; http://www.triticarte.com.au). Markers with a minor allele frequency lower than 0.05 were not included.

#### Wheat data set WHE5:

This data set is described by López-Cruz *et al.* (2015) and includes 807 wheat lines evaluated in five environments using an alpha-lattice design with three replicates in each environment at CIMMYT’s wheat breeding station at Cd. Obregon, Mexico. The environments were three irrigation regimes (0i = zero irrigation, 2i = two irrigations, and 5i = five irrigations), two planting systems (B = bed planting and F = flat planting) and two different planting dates (N = normal and L = late).

Genotypic data consisted of genotyping-by-sequencing (GBS) data, and markers with a minor allele frequency (MAF) lower than 0.05 were removed. After editing the missing markers, a total of 14,217 GBS markers were available for analyzing this data set.

#### Availability of the phenotypic and genotypic experimental data:

Sousa *et al.* (2017) describe the two maize data sets and Cuevas *et al.* (2017) give details of the two wheat data sets. The two maize data sets, HEL and USP, can be downloaded from the link http://hdl.handle.net/11529/10887, whereas the two wheat data sets can be found at the link http://hdl.handle.net/11529/10710, from where DATASET1.Wheat_GY.Rdata (Wheat data set WHE1) and DATASET5.Wheat_GY.Rdata (Wheat data set WHE5) were obtained.

### Statistical models

The components of the 8 basic models are summarized in Table 1 and their full descriptions are given below and in Appendix 1. They include an overall mean (μ) and the fixed effects of the environments (other effects can be incorporated) modeled with the incident matrix $Z_{E}$ and one vector of fixed effects $\beta_{E}$ for each environment. For the first group of six models (MM, MMl MDs, MDsl, MDe, and MDel), it is assumed that their genetic random components ***g*** have a normal distribution with mean zero and a variance-covariance structure comprising a known matrix K generated from markers (and computed using the GB or GK methods) multiplied by an unknown scaled parameter (variance component). Also 4 models in this group had different forms for modeling the G×E, MDs and MDe, with a variance-covariance structure constructed by the Hadamard product of the corresponding matrices and incorporating (or not) the random intercepts (l).

**Table 1:** Components of the 8 models included in this study. Each of these models is fitted with the linear kernel (GB) and the Gaussian kernel (GK)

Model	General mean	Fixed environmental effect	Main genetic effect of line across environments	Genotype × environment interaction (G×E)	Random intercept of the lines	Unstructured G×E	Random residual
MM	μ1	$Z_{E}\beta_{E}$	$Z_{g}g$				ε ($\sigma^{2})$
MMl	μ1	$Z_{E}\beta_{E}$	$Z_{g}g$		$Z_{g}l$ ($\sigma_{l}^{2})$		ε ($\sigma^{2})$
MDs	μ1	$Z_{E}\beta_{E}$	$Z_{g}g$	ge ($\sigma_{ge}^{2}$)			ε ($\sigma^{2})$
MDsl	μ1	$Z_{E}\beta_{E}$	$Z_{g}g$	ge ($\sigma_{ge}^{2}$)	$Z_{g}l$ ($\sigma_{l}^{2})$		ε ($\sigma^{2})$
MDe	μ1	$Z_{E}\beta_{E}$	$Z_{g}g$	$g_{E}$ ($\sigma_{g_{E_{j}}}^{2}$for each environment)			ε ($\sigma^{2})$
MDel	μ1	$Z_{E}\beta_{E}$	$Z_{g}g$	$g_{E}$ ($\sigma_{g_{E_{j}}}^{2}$for each environment)	$Z_{g}l$ ($\sigma_{l}^{2})$		ε ($\sigma^{2})$
MUC	μ1	$Z_{E}\beta_{E}$		u ($U_{E}⊗K)$			ε (Σ⊗I)
MUCf	μ1	$Z_{E}\beta_{E}$		u ($U_{E}⊗K)$		f ($F_{E}⊗I)$	ε (Σ⊗I)

A second group of models (MUC) considers that their random components have a normal distribution with zero mean and a variance-covariance structure modeled by the Kronecker product of a matrix with unknown covariances among environments multiplied by a known K (computed using the GB or GK methods) and incorporating (or not) the random intercepts (***f***).

#### The multi-environment main genotypic effect model (MM):

Model MM (1) (Appendix 1) is equivalent to the across-environment model of Jarquín *et al.* (2014) and when in the distribution of the random genetic effects g is used in model MM, $K=\frac{XX^{’}}{p}$ is used in the covariance (de los Campos *et al.* 2013; VanRaden 2007, 2008); the model is the GBLUP across environments (MM-GB), where X is the standardized matrix of molecular markers for the individuals of order n×p, where p is the number of markers.

However, markers can have a more complex function than the linear GBLUP. For example, the Gaussian kernel (GK) function (Cuevas *et al.* 2016) is computed as $K\left(x_{i},x_{i`}\right)=\text{exp}(-hd_{ii`}^{2})$, where $d_{ii`}$ is the Euclidean distance between the i^th^ and $i^{’\text{th}}\;\left(i=1,\ldots ,\;n_{j}\right)$ individuals given by the markers; h>0 is the bandwidth parameter that controls the rate of decay of K values (de los Campos *et al.* 2009; Pérez-Rodríguez *et al.* 2012; Pérez-Elizalde *et al.* 2015; Cuevas *et al.* 2016). In this work, GK is $K\left(x_{i},x_{i`}\right)=\text{exp}(-hd_{ii`}^{2}/\text{median}(d_{ii`}^{2})$), where h=1 and the median of the distances is used as a scaling factor (Crossa *et al.* 2010). When in the distribution of the random genetic effects g of the MM model (1) is used with $K\left(x_{i},x_{i`}\right)=\text{exp}(-hd_{ii`}^{2}/\text{median}(d_{ii`}^{2})$), in the covariance the model is the Gaussian kernel across environments (MM-GK) (Sousa *et al.* 2017).

The genetic variation between lines that is not explained by g in (1) (Appendix 1) can be captured by the random vector l that is considered a random intercept for each line; thus when random effects l are added, model MM becomes model MMl

$$y=\mu 1+Z_{E}\beta_{E}+Z_{g}g+Z_{g}l+\varepsilon$$

where the random intercepts $l∼N(0,\sigma_{l}^{2}I)$ with I being the identity matrix of size n×n, and $\sigma_{l}^{2}$ the variance component that indicates the influence of l; the incidence matrix $Z_{g}$ connects the genotypes to the phenotypes. As in MM, the kernel matrix K of the random effect ***g*** of model MMl can be fitted with GBLUP (MMl-GB) or with Gaussian kernel (MMl-GK).

#### The multi-environment single variance genotype × environment interaction deviation model (MDs):

Model (2) (Appendix 1) (MDs) adds to model (1) the random interaction effect of the environments with the genetic information of the lines ($ge_{ij}$). When the random component l is added to model (2), the MDs model becomes MDsl:

$$y=\mu 1+Z_{E}\beta_{E}+Z_{g}g+\mathrm{ge}+Z_{g}l+\varepsilon$$

Each environment matrix ***K*** (Appendix 1) of models MDs and MDsl can be fitted with a linear kernel (MDs-GB, MDsl-GB) or a Gaussian kernel (MDs-GK, MDsl-GK).

#### Multi-environment environment-specific variance genotype × environment deviation model (MDe):

The environment-specific variance genotype × environment deviation model (MDe) (López-Cruz *et al.* 2015) differs from MDs on how the interaction component is considered; ***g*** is the main genetic effect across environments and $g_{E}$ is the specific genetic effect in each environment. When the random component l is added to (3) (Appendix 1), the MDe model becomes MDel:

$$y=\mu 1+Z_{E}\beta_{E}+Z_{g}g+g_{E}+Z_{g}l+\varepsilon$$

where matrices ***K*** for **g** and ***K_E_*** for $g_{E}$ of models MDe and MDel can be fitted with a linear kernel (MDe-GB, MDel-GB) or with a Gaussian kernel (MDe-GK, MDel-GK).

#### Multi-environment With unstructured variance-covariance (MUC):

This model considers that there is a genetic correlation between environments that can be modeled with matrices of order m×m (where *m* denotes the environment) (Cuevas *et al.* 2017). The MUC is expressed as

$$y=\mu 1+Z_{E}\beta_{E}+u+\varepsilon$$

where $y={\left(y_{1},\ldots ,y_{j},\ldots y_{m}\right)}^{\prime }\;$is a vector with the observation $y_{j}$ belonging to the *j*^th^ environment (j=1,…,m), each of the same size (n); the random vector $u={\left(u_{1},\ldots ,u_{j},\ldots u_{m}\right)}^{\prime }$ is the vector of genetic values, and $\varepsilon ={\left(\varepsilon_{1},\ldots ,\varepsilon_{j},\ldots \varepsilon_{m}\right)}^{\prime }$the vector of random errors both assumed normally distributed with u $∼N(0,U_{E}⊗K)$ and **ε∼N(0,Σ⊗I),** where **⊗** is the Kronecker product.

The variance-covariance matrix of u is the Kronecker product of one unstructured matrix with information between environments $\left(U_{E}\right)$ that needs to be estimated and another known matrix with information between the lines based on Kmarkers(computed using the GB or GK methods). Then the m×m matrix $U_{E\;}$is

$$U_{E}=\left[\;\begin{array}{ccc} \sigma_{u_{1}}^{2} & \ldots & \begin{array}{ccc} \sigma_{u_{1}u_{j}} & \;\ldots & \sigma_{u_{1}u_{m}}\; \end{array}\\ \vdots & \ddots & \begin{array}{ccc} \vdots & \ddots & \;\;\;\;\;\;\vdots \; \end{array}\\ \begin{array}{c} \sigma_{u_{j}u_{1}}\\ \vdots \\ \sigma_{u_{m}u_{1}} \end{array} & \begin{array}{c} \ldots \\ \ddots \\ \ldots \end{array} & \begin{array}{ccc} \begin{array}{c} \sigma_{u_{j}}^{2}\\ \;\vdots \\ \sigma_{u_{m}u_{j}} \end{array} & \begin{array}{c} \ldots \\ \ddots \\ \ldots \end{array} & \begin{array}{c} \;\sigma_{u_{j}u_{m}}\;\\ \vdots \\ \sigma_{u_{m}}^{2} \end{array} \end{array} \end{array}\right]$$

where the *j^th^* diagonal element is the genetic variance $\sigma_{u_{j}}^{2}$within the *j^th^* environment, and the off-diagonal elements are the genetic covariances $\sigma_{u_{j}u_{j’}}$between environments *j* and *j*’. For a large number of environments, a factor analytical model usually performs better than the unstructured model (Burgueño *et al.* 2012; Oakey *et al.* 2016). Furthermore, matrix Σ is an error diagonal matrix of order m×m, *i.e.*, Σ**=***diag*($\sigma_{\varepsilon_{1}}^{2},\ldots .,\sigma_{\varepsilon_{m}}^{2}$).

#### Multi-environment With un-structured variance-covariance and random intercepts (MUCf):

The MUC model can be extended by adding an extra variability to account for genetic variance among individuals across environments, that is, by adding the random vector ***f*** (Cuevas *et al.* 2017). Therefore, the extension of the previous random linear model is

$$y=\mu 1+Z_{E}\beta_{E}+u+f+\varepsilon$$

where $f={\left(f_{1},\ldots ,f_{j},\ldots ,f_{m}\right)}^{\prime }$ with the random vectors $f_{j}\;$being independent of $u_{j}$ and normally distributed $f∼N(0,F_{E}⊗I)$. Matrix $F_{E,}\;$is unstructured and captures genetic variance-covariance effects between the individuals across environments that were not captured by the $U_{E}$ matrix; matrix $F_{E}$ can be expressed as

$$F_{E}=\left[\;\begin{array}{ccc} \sigma_{f_{1}}^{2} & \ldots & \begin{array}{ccc} \sigma_{f_{1}f_{j}} & \;\ldots & \sigma_{f_{1}f_{m}}\; \end{array}\\ \vdots & \ddots & \begin{array}{ccc} \vdots & \ddots & \;\vdots \; \end{array}\\ \begin{array}{c} \sigma_{f_{j}f_{1}}\\ \vdots \\ \sigma_{f_{m}f_{1}} \end{array} & \begin{array}{c} \ldots \\ \ddots \\ \ldots \end{array} & \begin{array}{ccc} \begin{array}{c} \sigma_{f_{j}}^{2}\\ \;\vdots \\ \sigma_{f_{m}f_{j}} \end{array} & \begin{array}{c} \ldots \\ \ddots \\ \ldots \end{array} & \begin{array}{c} \;\sigma_{f}\;\\ \vdots \\ \sigma_{f_{m}}^{2} \end{array} \end{array} \end{array}\right]$$

where the *j^th^* diagonal element of the m×m matrix $F_{E}$ is the genetic environmental variance $\sigma_{f_{j}}^{2}$ within the *j^th^* environment, and the off-diagonal element is the genetic covariance $\sigma_{f_{j}f_{j’}}$ between environments *j* and *j*’. Similar to the previous cases, models MUC and MUC***f*** can be fitted using GB or GK kernels to generate the four model-method MUC-GB,MUC-GK, MUC***f***-GB, MUC***f***-GK.

#### Model implementation and random cross-validation for assessing prediction accuracy in the four data sets:

For the two maize data sets, models MM-GB, MM-GK, MDs-GB, MDs-GK, MDe-GB, and MDe-GK were fitted with the new software BGGE (Granato *et al.* 2017). Models MMl-GB, MMl-GK, MDsl-GB, MDsl-GK, MDel-GB, and MDel-GK were also fitted with BGGE with the same random partitions used by Sousa *et al.* (2017) to make results comparable for random-cross-validation 1 (CV1) and random cross-validation 2 (CV2) (Burgueño *et al.* 2012). Models MUC***f*** and MUC of Cuevas *et al.* (2017) were fitted using the software MTM (de los Campos and Grüneberg 2016) with the GB and GK kernel methods and with the same random partitions used for the 12 model-method combinations previously defined for random cross-validations CV1 and CV2. A fivefold random cross-validation was used assigning 80% of the observations to the training sets and 20% to the testing (validation) set. However, most of the results and discussion focus on cross-validation CV2. The two wheat data sets were fitted with the 12 model-method combinations (models MMl-GB, MMl-GK, MDsl-GB, MDsl-GK, MDel-GB, MDel-GK, MM-GB, MM-GK, MDs-GB, MDs-GK, MDe-GB, MDe-GK) using the BGGE software of Granato *et al.* (2017).

Two random cross-validations (CV1 and CV2) were generated; CV1 attempts to mimic a situation where a set of lines were never evaluated in a set of environments, whereas CV2 mimics a sparse testing scheme where some lines were evaluated in some environments but not in others. Results based on CV2 are shown in the main text, tables and figures. Results of random cross-validation CV1 are given in Tables S1-S4 of Appendix 2. To implement the proposed 12 model-method combinations, 50 random partitions were performed with 80% of the lines used for training and the remaining 20% of the lines used for testing. The metric for measuring the performance of prediction accuracy was the Pearson correlation calculated between the observed and predicted values of the testing sets.

## Results

The results are given in four sections, one for each data set. In each section, we provide the results of the variance component estimates and the prediction accuracy for each of the 12 model-method combinations.

### Maize data set HEL

This maize data set has a total of 452 maize hybrids with a different number in each of the five sites ($n_{IP}=$ 247, $n_{NM}=$ 330, $n_{PM}=$ 452, $n_{SE}=$ 367, $n_{SO}=$ 330). The sample phenotypic correlations among locations are positive with intermediate-to-low values, where location SE has low correlations with all the other locations, and locations NM, IP, and PM show relatively high correlations with the other locations (Table A1, Appendix 3).

Models without the random component l always show a lower residual variance component in the GK models than in the GB models; for example, for model MDs-GK, $\sigma^{2}$ = 0.278 and for MDs-GB, $\sigma^{2}$ = 0.591 (Table 2). However, when the models include l, these differences become smaller; for example, for MDsl-GK, **$\sigma^{2}$** = 0.277 and for MDsl-GB, **$\sigma^{2}$** = 0.368, indicating that for method GB, the random component l explains the variation of the observations better, whereas for GK, including l does not have much influence on the residual. This is also reflected in the small value of $\sigma_{l}^{2}$ = 0.013 for MDsl-GK as compared with $\sigma_{l}^{2}$ = 0.243 for MDsl-GB.

**Table 2:** MAIZE HEL data set. Estimated variance components for the multi-environment models, main genotypic effect model (MM), single variance G×E deviation model (MDs) and environment-specific variance G×E deviation model (MDe) with two kernels, GBLUP (GB) and Gaussian (GK), with l and without l, for grain yield (standard deviation in parentheses)

Variance Component^*^	MMl-GK	MMl-GB	MDsl-GK	MDsl-GB	MDel**-**GK	MDel**-**GB	MM- GK	MM- GB	MDs-GK	MDs- GB	MDe-GK	MDe- GB
$\sigma^{2}$	0.581 (0.02)	0.594 (0.02)	0.277 (0.02)	0.368 (0.02)	0.246 (0.02)	0.368 (0.02)	0.582 (0.02)	0.749 (0.03)	0.278 (0.02)	0.591 (0.02)	0.247 (0.02)	0.592 (0.02)
$\sigma_{g}^{2}$	0.795 (0.1)	0.228 (0.06)	0.871 (0.11)	0.172 (0.06)	0.88 (0.12)	0.186 (0.06)	0.821 (0.1)	0.356 (0.08)	0.938 (0.1)	0.370 (0.08)	0.931 (0.1)	0.390 (0.09)
$\sigma_{ge}^{2}$	—	—	0.53 (0.06)	0.256 (0.03)	—	—	—	—	0.525 (0.06)	0.188 (0.03)	—	—
$\sigma_{IP}^{2}$	—	—	—	—	0.376 (0.1)	0.257 (0.08)	—	—	—	—	0.372 (0.1)	0.237 (0.08)
$\sigma_{NM}^{2}$	—	—	—	—	0.778 (0.19)	0.197 (0.08)	—	—	—	—	0.769 (0.2)	0.076 (0.06)
$\sigma_{PM}^{2}$	—	—	—	—	0.374 (0.08)	0.297 (0.08)	—	—	—	—	0.370 (0.09)	0.259 (0.08)
$\sigma_{SE}^{2}$	—	—	—	—	1.135 (0.2)	0.385 (0.11)	—	—	—	—	1.143 (0.21)	0.255 (0.09)
$\sigma_{SO}^{2}$	—	—	—	—	0.688 (0.16)	0.215 (0.08)	—	—	—	—	0.688 (0.16)	0.158 (0.07)
$\sigma_{l}^{2}$	0.008 (0.01)	0.201 (0.03)	0.013 (0.01)	0.243 (0.03)	0.014 (0.01)	0.244 (0.03)	—	—	—	—	—	—

* Locations are: IP: Ipiaçú-MG, NM: Nova Mutum-MT, PM: Pato de Minas-MG, SE: Sertanópolis-PR, and SO: Sorriso-MT.

The size of the genetic component, $\sigma_{g}^{2}$, is always much higher for MM-GK, MDs-GK, and MDe-GK than for models with the GB method. For models MMl, MDsl, and MDel, the sum of **$\sigma_{g}^{2}$** and $\sigma_{l}^{2}$ is higher than the component $\sigma_{g}^{2}$ for models MM, MDs, and MDe. For example, for model MMl-GB, $\sigma_{g}^{2}+\sigma_{l}^{2}$ is 0.429, whereas for model MM-GB, $\sigma_{g}^{2}$ is 0.356; MDsl-GB summation $\sigma_{g}^{2}+\sigma_{l}^{2}$ is 0.415 *vs.* MDs-GB with $\sigma_{g}^{2}$ = 0.370, and for model MDel-GB $\sigma_{g}^{2}+\sigma_{l}^{2}$= 0.430 *vs.* MDs-GB with $\sigma_{g}^{2}$ = 0.370. The variance explained by the G×E of MDs, $\sigma_{ge}^{2}$, is higher for GK than for GB and slightly higher for models with the random component l than for models without **l**. The variance components for the specific environments show increases in MDel-GK compared to MDel-GB, and in MDe-GK compared to MDe-GK (Table 2).

**Figure 1:**
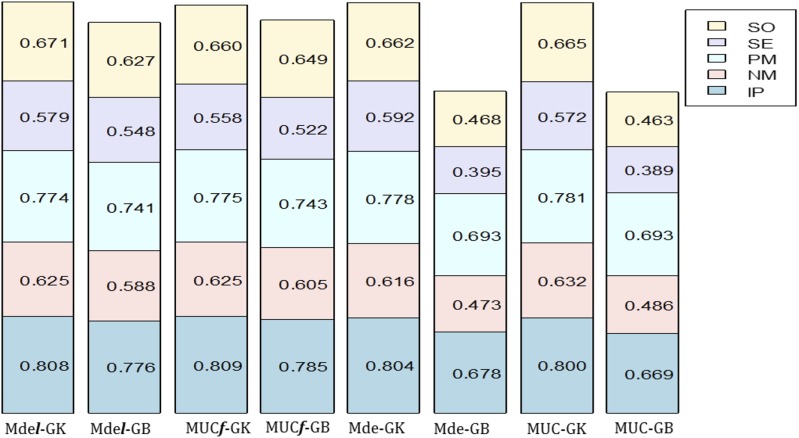
Plot of the prediction accuracy using Pearson’s correlation for each of the 5 locations (SO, SE, PM, NM, and IP) of maize data set HEL for the proposed models MDe***l***-GK, MDe***l***-GB, MUC***f***-GK, MUC***f***-GB, MDe-GK, MDe-GB, MUC-GK, and MUC-GB.

Models including the random component l with GK did not improve the prediction accuracy of the locations as compared with the prediction accuracy of models without l with GK (Table 3 and Figure 1); however, models with l had consistently higher prediction accuracies than models with GB. In all cases, MMl showed lower prediction accuracy than models with G×E (MDsl and MDel). Similarly, model MM had lower prediction accuracies than models that incorporate G×E (MDs and MDe). These differences are smaller for locations that had higher sample phenotypic correlations with other locations than for locations with low phenotypic correlations. For example, location NM had prediction accuracies of 0.569, 0.589, and 0.588 for models MMl-GB, MDsl-GB, and MDel-GB, respectively, whereas location SE with low sample phenotypic correlations among locations had prediction accuracies of 0.372, 0.544, and 0.548 for models MMl-GB, MDsl-GB, and MDel-GB, respectively.

**Table 3:** Maize HEL data set. Mean Pearson’s correlation (50 partitions) of each location for random cross-validation CV2, for the multi-environment models, main genotypic effect model (MM), single variance G×E deviation model (MDs), environment-specific variance G×E deviation model (MDe), multi-environment unstructured covariance models (MUC and MUC*f*) with two kernels, GBLUP (GB) and Gaussian kernel (GK) for grain yield with the proposed random effect l and without the random effect l (standard deviation in parentheses)

	Proposed models with random effects l and ***f***							
Location*	MMl-GK	MMl-GB	MDsl-GK	MDsl-GB	MDel**-**GK	MDel**-**GB	MUC***f***-GK	MUC***f***-GB
IP	0.596 (0.1)	0.577 (0.11)	0.802 (0.05)	0.778 (0.05)	0.808 (0.05)	0.776 (0.05)	0.809 (0.04)	0.785 (0.05)
NM	0.601 (0.09)	0.569 (0.09)	0.614 (0.08)	0.589 (0.08)	0.625 (0.08)	0.588 (0.08)	0.625 (0.06)	0.605 (0.07)
PM	0.643 (0.06)	0.589 (0.05)	0.776 (0.04)	0.733 (0.05)	0.774 (0.05)	0.741 (0.04)	0.775 (0.03)	0.743 (0.05)
SE	0.42 (0.09)	0.372 (0.11)	0.586 (0.08)	0.544 (0.08)	0.579 (0.07)	0.548 (0.08)	0.558 (0.08)	0.522 (0.1)
SO	0.544 (0.11)	0.523 (0.11)	0.673 (0.05)	0.639 (0.06)	0.671 (0.06)	0.627 (0.08)	0.66 (0.06)	0.649 (0.06)
	Proposed models without random effects l and ***f***							
Location	MM-GK	MM-GB	MDs-GK	MDs-GB	MDe-GK	MDe**-**GB	MUC-GK	MUC-GB
IP	0.595 (0.08)	0.51 (0.11)	0.807 (0.04)	0.683 (0.08)	0.804 (0.05)	0.678 (0.07)	0.800 (0.05)	0.669 (0.09)
NM	0.601 (0.08)	0.469 (0.10)	0.627 (0.08)	0.472 (0.08)	0.616 (0.09)	0.473 (0.11)	0.632 (0.07)	0.486 (0.08)
PM	0.645 (0.06)	0.584 (0.08)	0.776 (0.04)	0.697 (0.05)	0.778 (0.04)	0.693 (0.05)	0.781 (0.04)	0.693 (0.04)
SE	0.427 (0.1)	0.296 (0.1)	0.591 (0.07)	0.39 (0.08)	0.592 (0.08)	0.395 (0.1)	0.572 (0.07)	0.389 (0.09)
SO	0.558 (0.07)	0.396 (0.11)	0.666 (0.06)	0.466 (0.08)	0.662 (0.06)	0.468 (0.09)	0.665 (0.07)	0.463 (0.10)

* Locations are: IP: Ipiaçú-MG, NM: Nova Mutum-MT, PM: Pato de Minas-MG, SE: Sertanópolis-PR, and SO: Sorriso-MT.

All models with kernel GK had higher prediction accuracies (with and without the random component l) than models with kernel GB (Table 3 and Figure 1). However, these differences are lower for models that include the random component l (Table 3). For example, for location SO, the prediction accuracies for models MDsl-GK and MDsl-GB were 0.673 and 0.639, respectively, whereas for MDs-GK and MDs-GB, the mean prediction accuracies were 0.666 and 0.466, respectively. Comparing models with kernel GB, with and without l, the predictions are always higher when the model includes **l** than when the model excludes l; for example, for location IP, the mean prediction accuracies were 0.778 and 0.683 for MDsl-GB and MDs-GB, respectively (Table 3). Note that the variance component of the random effect l **$\sigma_{l}^{2}$** was 0.243 for model MDsl-GB (Table 2). Furthermore, model 3 from Cuevas *et al.* (2017) with the unstructured variance-covariance component ***f*** or model 2 without ***f*** did not show any clear superiority, in terms of mean prediction accuracy, over models MDsl and MDel and MDs and MDe with GK and GB (Table 3 and Figure 1).

Random cross-validation CV1 decreased the prediction accuracy as compared with results achieved for CV2 (Table S1, Appendix 2); the trends and patterns of the prediction accuracy of the locations between models and methods are similar to those found for CV2, including those found for models MUC and MUC***f***.

In summary, results from maize data HEL indicated that models with the random component l with GK including G×E (MDsl-GK and MDel-GK) show similar mean prediction accuracy as models excluding the random component l. However, this did not occur with GB models where including the random component l increased the prediction accuracy for all 5 locations. Prediction accuracy using GK was always higher than using GB with or without the random component l. Also, the differences between the models with and without **l** and between GK and GB were smaller for locations that had higher sample phenotypic correlations with other locations. Finally, the differences in prediction accuracy were negligible between the proposed models including G×E with GK and GB and with and without the random effect l and models MUC***f*** and MUC for all locations.

### Maize data set USP

This maize data set is comprised of 739 maize hybrids with different numbers of lines in each of the four sites ($n_{P-LN}=$ 731, $n_{P-IN}=$ 732, $n_{A-LN}=$ 731, $n_{A-IN}=$ 737). Locations P-IN and A-IN had relatively high correlations with the other locations, whereas A-LN had low ones (Table A1, Appendix 3). The residual variance components for GK are smaller than those for GB for models MM, MDs and MDe; for instance, MM-GK had $\sigma^{2}$ = 0.589 while MM-GB had $\sigma^{2}$ = 0.854. Similarly, the residual variance components for MDsl and MDel with GK are lower than for MDsl and MDel with GB. The variance components of the random intercept ($\sigma_{l}^{2}$) of GK methods are not negligible (as in data set HEL) and are always lower than for the corresponding GB methods (Table 4).

**Table 4:** Maize USP data set. Estimated variance components for the multi-environment models, main genotypic effect model (MM), single variance G×E deviation model (MDs) and environment-specific variance G×E deviation model (MDe) with two kernels, GBLUP (GB) and Gaussian kernel (GK), with l and without l for grain yield (standard deviation in parentheses)

Variance Component^*^	MMl-GK	MMl-GB	MDsl-GK	MDsl-GB	MDel**-**GK	MDel**-**GB		MM- GK	MM- GB	MDs-GK	MDs- GB	MDe-GK	Mde- GB
$\sigma^{2}$	0.547 (0.02)	0.548 (0.02)	0.49 (0.02)	0.503 (0.02)	0.487 (0.02)	0.503 (0.02)		0.589 (0.02)	0.854 (0.02)	0.538 (0.02)	0.834 (0.02)	0.534 (0.02)	0.833 (0.02)
$\sigma_{g}^{2}$	0.371 (0.09)	0.175 (0.05)	0.343 (0.09)	0.164 (0.05)	0.362 (0.1)	0.165 (0.05)		1.899 (0.18)	0.214 (0.06)	2.012 (0.18)	0.209 (0.06)	2.026 (0.19)	0.206 (0.06)
$\sigma_{ge}^{2}$	—	—	0.091 (0.02)	0.045 (0.01)	—	—		—	—	0.077 (0.02)	0.029 (0.01)	—	—
$\sigma_{P-LN}^{2}$	—	—	—	—	0.159 (0.09)	0.055 (0.03)		—	—	—	—	0.162 (0.07)	0.031 (0.03)
$\sigma_{P-IN}^{2}$	—	—	—	—	0.104 (0.06)	0.048 (0.03)		—	—	—	—	0.107 (0.05)	0.034 (0.02)
$\sigma_{A-LN}^{2}$	—	—	—	—	0.093 (0.07)	0.046 (0.03)		—	—	—	—	0.073 (0.05)	0.038 (0.03)
$\sigma_{A-IN}^{2}$	—	—	—	—	0.084 (0.06)	0.063 (0.03)		—	—	—	—	0.05 (0.04)	0.052 (0.03)
$\sigma_{l}^{2}$	0.279 (0.03)	0.336 (0.03)	0.296 (0.03)	0.349 (0.03)	0.294 (0.03)	0.349 (0.03)		—	—	—	—	—	—

* Locations are: Anhumas ideal N (A-IN), Anhumas low N (A-LN), Piracicaba ideal N (P-IN) and Piracicaba low N (P-LN)

The estimated genetic variance components $\sigma_{g}^{2}$ for GB in models MM, MDs and MDe were 0.214, 0.209, and 0.206, respectively (Table 4), increasing the genetic environmental stability ($\sigma_{g}^{2}+\sigma_{l}^{2})\;$of models MMl-GB, MDsl-GB, and MDel-GB to 0.511, 0.513, and 0.514, respectively. The specific components for each environment of models MDe-GB and MDel-GB were negligible. The variance component ($\sigma_{ge}^{2}$) of the G×E models MDs and MDsl for GB and GK was also negligible.

**Figure 2:**
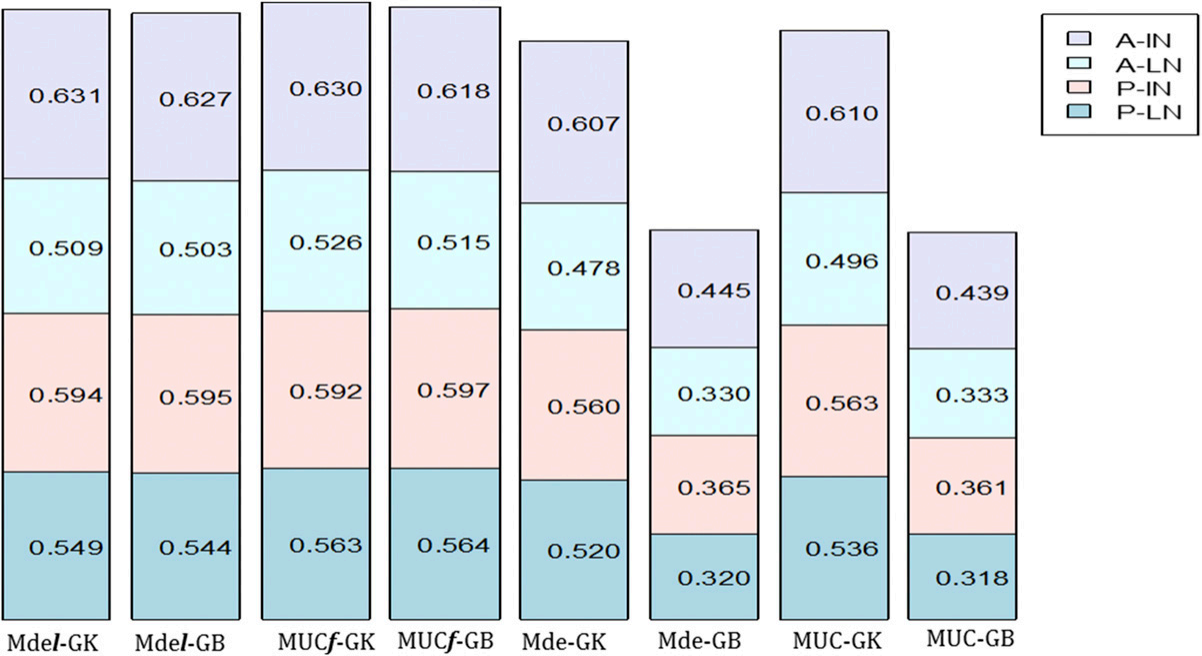
Plot of the prediction accuracy using Pearson’s correlation for each of the 4 environments (P-LN, P-IN, A-LN, A-IN) of maize data set USP for the proposed models MDe***l***-GK, MDe***l***-GB, MUC***f***-GK, MUC***f***-GB, MDe-GK, MDe-GB, MUC-GK, and MUC-GB.

In general, models with l-GB had similar prediction accuracy as models with l-GK, whereas the increase in prediction accuracy of models without l-GK over models with GB is clear. For example, for P-LN, models MDsl-GK and MDsl-GB had prediction accuracies of 0.545 and 0.546, respectively, whereas for MDs-GK and MDs-GB, the prediction accuracies were 0.524 and 0.325 (Table 5 and Figure 2). Models with l-GB showed significant improvement in prediction accuracy compared to models GB without l; for example, for location P-IN, the mean prediction accuracies of MDsl-GB and MDs-GB were 0.591 and 0.368, respectively (due to the influence of $\sigma_{l}^{2}$ = 0.349 for model MDsl-GB; see Table 4). All models with GK with the random intercept l and with high values of **$\sigma_{l}^{2}\;$**gave higher prediction accuracies than GK models without l. There are no clear differences between model MUC***f*** and the proposed model with the random component l  with GK and GB in all the locations. Similar results were found for model MUC when compared to models without l. For this data set, results from CV1 (Table S2, Appendix 2) were all similar and lower than those obtained for CV2.

**Table 5:** Maize USP data set. Mean Pearson’s correlation (50 partitions) of each environment for random cross-validation CV2, for the multi-environment models, main genotypic effect model (MM), single variance G×E deviation model (MDs), environment-specific variance G×E deviation model (MDe), multi-environment unstructured covariance models (MUC and MUCf) with two kernels, GBLUP (GB) and Gaussian kernel (GK) for grain yield with the proposed random effect l and without the random effect l (standard deviation in parentheses)

	Proposed models with random effects l and ***f***							
Environment*	MMl-GK	MMl-GB	MDsl-GK	MDsl-GB	MDel**-**GK	MDel**-**GB	MUC***f***-GK MUC***f***-GB	
P-LN	0.525 (0.07)	0.521 (0.06)	0.545 (0.07)	0.546 (0.07)	0.549 (0.05)	0.544 (0.08)	0.563 (0.06)	0.564 (0.06)
P-IN	0.575 (0.05)	0.566 (0.06)	0.593 (0.05)	0.591 (0.06)	0.594 (0.05)	0.595 (0.04)	0.592 (0.06)	0.597 (0.05)
A-LN	0.493 (0.06)	0.493 (0.07)	0.508 (0.06)	0.515 (0.06)	0.509 (0.05)	0.503 (0.05)	0.526 (0.05)	0.515 (0.06)
A-IN	0.603 (0.05)	0.599 (0.06)	0.627 (0.05)	0.629 (0.06)	0.631 (0.05)	0.627 (0.06)	0.630 (0.05)	0.618 (0.06)
	Proposed models without random effects l and ***f***							
Environment	MM-GK	MM-GB	MDs-GK	MDs-GB	MDe-GK	MDe**-**GB	MUC-GK MUC-GB
P-LN	0.50 (0.06)	0.315 (0.06)	0.524 (0.07)	0.325 (0.06)	0.52 (0.06)	0.32 (0.05)	0.536 (0.07)	0.318 (0.06)
P-IN	0.53 (0.05)	0.358 (0.06)	0.554 (0.05)	0.368 (0.05)	0.56 (0.05)	0.365 (0.06)	0.563 (0.05)	0.361 (0.07)
A-LN	0.463 (0.07)	0.332 (0.07)	0.476 (0.06)	0.334 (0.07)	0.478 (0.06)	0.33 (0.07)	0.496 (0.06)	0.333 (0.06)
A-IN	0.584 (0.06)	0.438 (0.07)	0.612 (0.05)	0.447 (0.05)	0.607 (0.04)	0.445 (0.06)	0.61 (0.05)	0.439 (0.06)

* Environments are: Anhumas ideal N (A-IN), Anhumas low N (A-LN), Piracicaba ideal N (P-IN) and Piracicaba low N (P-LN).

In summary, results from maize data USP indicate that models with the random component l (MDsl-GK and MDel-GK) show higher mean prediction accuracy than models without l and using the linear kernel GB. The G×E variance component of models MDs and MDsl with GK and GB had negligible $\sigma_{ge}^{2}$, indicating less complex G×E than that found for maize data set HEL. The differences in the mean prediction accuracy between the proposed models with or without the random effect l and models MUC***f*** and MUC are small for models with GK and not clearly superior to the proposed models with GB.

### Wheat data set WHE1

For this data set, environment E1 had negative correlations with the other environments (E2-E4), whereas environments E2-E4 had high correlations among themselves (Table A1, Appendix 3). Models with GK fitted the WHE1 data better than models with kernel GB (low residual variances of GK models as compared to GB models). Also, models with random component l had lower residual variance components than models without l. As opposed to the previous two maize data sets, where the magnitude of the variance components determines the prediction ability, the presence of environments with negative correlations with other environments makes interpreting the variance components in relation to their predictive ability not as straightforward as in the previous two data sets (Table 6). For example, models MMl and MM with GK and GB had estimates of the random error variance that were much higher (∼0.8) than those of the other models; thus the prediction accuracy of these models is expected to be low for at least the environments with negative correlations.

**Table 6:** Wheat WHE1 data set. Estimated variance components for the multi-environment models, main genotypic effect model (MM), single variance G×E deviation model (MDs) and environment-specific variance G×E deviation model (MDe) with two kernels, GBLUP (GB) and Gaussian (GK), with l and without l for grain yield (standard deviation in parentheses)

Variance component^*^	MMl-GK	MMl-GB	MDsl-GK	MDsl-GB	MDel**-**GK	MDel**-**GB		MM- GK	MM- GB	MDs-GK	MDs- GB	MDe-GK	Mde- GB
$\sigma^{2}$	0.805 (0.03)	0.81 (0.03)	0.388 (0.02)	0.471 (0.02)	0.416 (0.02)	0.479 (0.02)		0.812 (0.03)	0.824 (0.03)	0.462 (0.03)	0.551 (0.02)	0.471 (0.02)	0.533 (0.02)
$\sigma_{g}^{2}$	0.597 (0.11)	0.177 (0.04)	0.262 (0.14)	0.074 (0.04)	1.028 (0.2)	0.326 (0.06)		0.599 (0.1)	0.192 (0.03)	0.752 (0.14)	0.219 (0.05)	1.404 (0.17)	0.414 (0.06)
$\sigma_{ge}^{2}$	—	—	1.637 (0.16)	0.42 (0.05)	—	—		—	—	1.349 (0.15)	0.349 (0.04)	—	—
$\sigma_{1}^{2}$	—	—	—	—	3.356 (0.44)	1.058 (0.15)		—	—	—	—	3.026 (0.39)	0.868 (0.13)
$\sigma_{2}^{2}$	—	—	—	—	0.271 (0.16)	0.038 (0.03)		—	—	—	—	0.142 (0.07)	0.08 (0.03)
$\sigma_{3}^{2}$	—	—	—	—	0.382 (0.24)	0.031 (0.03)		—	—	—	—	0.135 (0.07)	0.076 (0.03)
$\sigma_{4}^{2}$	—	—	—	—	1.147 (0.24)	0.3 (0.08)		—	—		—	0.839 (0.22)	0.217 (0.06)
$\sigma_{l}^{2}$	0.014 (0.01)	0.024 (0.02)	0.101 (0.02)	0.107 (0.02)	0.077 (0.02)	0.09 (0.02)		—	—	—	—	—	

* Environments are 1, 2, 3, and 4.

The genetic variance component $\sigma_{g}^{2}$ varied for models MM-GB, MDs-GB, and MDe-GB (0.192, 0.219, and 0.414, respectively) as well as for the GK models (0.599, 0.752, and 1.404, respectively). The contribution of l measured in $\sigma_{l}^{2}$ was small for MDsl-GK and MDsl-GB (0.101 and 0.107) (Table 6) and negligible for the other models with l. On the other hand, the G×E interaction variance components $\sigma_{gE}^{2}$ for GK and GB are important (MDsl-GK $\sigma_{gE}^{2}$ = 1.637, MDsl-GB $\sigma_{gE}^{2}$ = 0.42; MDs-GK $\sigma_{gE}^{2}$ = 1.349, MDs-GB $\sigma_{gE}^{2}$ = 0.349) and much higher than in the two maize data sets. Models MDel-GK and MDel-GB showed high specific variance components for E1 (3.356 and 1.058, respectively) and for E4 (1.147 and 0.3) causing most of the interaction in this data set (these are the environments with the lowest sample correlations with the other environments) and contributed the least to genetic environmental stability.

**Figure 3:**
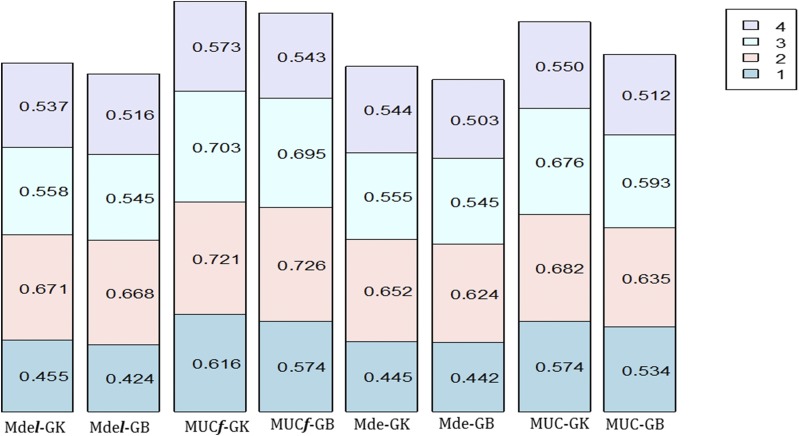
Plot of the prediction accuracy using Pearson’s correlation for each of the 4 environments (E1-E4) of wheat data set WHE1 for the proposed models, MDe***l***-GK, MDe***l***-GB, MUC***f***-GK, MUC***f***-GB, MDe-GK, MDe-GB, MUC-GK, and MUC-GB.

Models with G×E (MDs and MDe) had mean prediction accuracies higher than MM models with lower mean prediction accuracy in E1 and E4 as compared with E2 and E3 (Table 7 and Figure 3). The exceptions are models MM-GB and MM-GK, which had higher prediction accuracy than models MDs-GB and MDS-GB in E3. Models MDel-GK and MDe-GK had higher prediction accuracy than models MM, MDs and MDe with and without l for GB and GK in all locations, except MDe-GK in E1. However, in all cases and environments, models MUC***f*** and MUC had better prediction accuracies than all 12 genomic model-method combinations (Figure 3). Lower prediction accuracies were found for CV1 (Table S3, Appendix 2) than for CV2; however, the decrease in prediction accuracy of CV1 was lower than for the two wheat data sets.

**Table 7:** WHEAT WHE1 data set. Mean Pearson’s correlation (50 partitions) of each environment for random cross-validation CV2, for the multi-environment models, main genotypic effect model (MM), single variance G×E deviation model (MDs), environment-specific variance G×E deviation model (MDe), multi-environment unstructured covariance models (MUC and MUCf) with two kernels, GBLUP (GB) and Gaussian kernel (GK) for grain yield with the proposed random effect l and without the random effect l (standard deviation in parentheses)

	Proposed models with random effects l and ***f***							
Environment^*^	MMl-GK	MMl-GB	MDsl-GK	MDsl-GB	MDel**-**GK	MDel**-**GB	MUC***f***-GK MUC***f***-GB	
E1	−0.052 (0.06)	−0.048 (0.07)	0.458 (0.05)	0.422 (0.07)	0.455 (0.06)	0.424 (0.05)	0.616 (0.06)	0.574 (0.07)
E2	0.572 (0.04)	0.572 (0.05)	0.625 (0.03)	0.626 (0.05)	0.671 (0.04)	0.668 (0.04)	0.721 (0.04)	0.726 (0.04)
E3	0.486 (0.05)	0.482 (0.05)	0.50 (0.05)	0.473 (0.06)	0.558 (0.04)	0.545 (0.05)	0.703 (0.04)	0.695 (0.04)
E4	0.402 (0.06)	0.399 (0.05)	0.525 (0.05)	0.501 (0.06)	0.537 (0.05)	0.516 (0.05)	0.573 (0.06)	0.543 (0.06)
	Proposed models without random effecst l and ***f***							
Environment	MM-GK	MM-GB	MDs-GK	MDs-GB	MDe-GK	MDe**-**GB	MUC-GK MUC-GB
E1	−0.026 (0.06)	−0.024 (0.07)	0.478 (0.06)	0.458 (0.06)	0.445 (0.07)	0.442 (0.06)	0.574 (0.08)	0.534 (0.07)
E2	0.558 (0.05)	0.541 (0.05)	0.593 (0.05)	0.562 (0.04)	0.652 (0.04)	0.624 (0.04)	0.682 (0.06)	0.635 (0.05)
E3	0.486 (0.06)	0.481 (0.05)	0.47 (0.06)	0.457 (0.06)	0.555 (0.05)	0.545 (0.05)	0.676 (0.04)	0.593 (0.04)
E4	0.406 (0.05)	0.388 (0.06)	0.52 (0.04)	0.463 (0.05)	0.544 (0.05)	0.503 (0.05)	0.550 (0.06)	0.512 (0.07)

* Environments are, E1, E2, E3, and E4.

In summary, G×E for this data set is more complex than for the two previous maize data sets. This is expressed by higher values of $\sigma_{gE}^{2}$ (given by models MDsl and MDs) compared to those computed for the maize data sets, as well as the higher values of the variance components specific to environments ($\sigma_{1}^{2}$ and $\sigma_{4}^{2}$) compared to those computed for other environments in this data set, as well as in the maize data sets. For the 12 model-method combinations, the models with the highest prediction accuracy for the environments were MDel and MDe. However, models MU***f*** and MUC had the highest prediction accuracy for each environment and for both methods, GK and GB.

### Wheat data set WHE5

This data set has sample phenotypic correlations between environments that are close to zero or negative (Table A1, Appendix 3). Only one high phenotypic correlation was observed between environments 5iBN and 5iFN (0.546). Table 8 shows the high residual variance components of models MMl-GK, MMl-GB, MM-GK and MM-GB, whereas for models incorporating G×E (MDs and MDe with GK and GB and with and without l), the residual variance components were much smaller.

**Table 8:** WHEAT WHE5 data set. Estimated variance components for the multi-environment models, main genotypic effect model (MM), single variance G×E deviation model (MDs) and environment-specific variance G×E deviation model (MDe) with two kernels, GBLUP (GB) and Gaussian kernel (GK), with l and without l (**Sousa *et al.* 2017**), for grain yield (standard deviation in parentheses)

Variance component^*^	MMl-GK	MMl-GB	MDsl-GK	MDsl-GB	MDel**-**GK	MDel**-**GB		MM- GK	MM- GB	MDs-GK	MDs- GB	MDe-GK	Mde- GB
$\sigma^{2}$	0.879 (0.02)	0.883 (0.02)	0.001 (0.00)	0.269 (0.03)	0.001 (0.00)	0.248 (0.03)		0.88 (0.02)	0.884 (0.02)	0.001 (0.00)	0.282 (0.03)	0.002 (0.00)	0.267 (0.03)
$\sigma_{g}^{2}$	0.168 (0.02)	0.102 (0.01)	0.131 (0.01)	0.064 (0.03)	0.105 (0.02)	0.061 (0.03)		0.17 (0.02)	0.105 (0.01)	0.16 (0.02)	0.125 (0.02)	0.178 (0.02)	0.119 (0.02)
$\sigma_{ge}^{2}$	—	—	1.49 (0.03)	0.636 (0.05)	—	—		—	—	1.482 (0.04)	0.618 (0.05)	—	—
$\sigma_{0iFn}^{2}$	—	—	—	—	1.385 (0.07)	0.639 (0.07)		—	—	—	—	1.37 (0.07)	0.607 (0.06)
$\sigma_{2iBN}^{2}$	—	—	—	—	1.578 (0.08)	0.722 (0.08)		—	—	—	—	1.568 (0.08)	0.693 (0.08)
$\sigma_{5iBH}^{2}$	—	—	—	—	1.262 (0.07)	0.554 (0.06)		—	—	—	—	1.187 (0.07)	0.528 (0.06)
$\sigma_{5iBN}^{2}$	—	—	—	—	1.619 (0.08)	0.74 (0.08)		—	—	—	—	1.637 (0.09)	0.716 (0.07)
$\sigma_{5iFN}^{2}$	—	—	—	—	1.73 (0.09)	0.74 (0.08)		—	—	—	—	1.709 (0.09)	0.717 (0.08)
$\sigma_{l}^{2}$	0.003 (0.0)	0.004 (0.0)	0.014 (0.0)	0.043 (0.02)	0.02 (0.01)	0.039 (0.02)		—	—	—	—	—	—

* Environments are described by a sequence of codes: 0i, 2i and 5i denote the number of irrigation cycles; B/F denotes whether the planting system was ‘bed’ (B) or ‘flat’ (F); N/H denotes whether planting date was normal (N) or late (H, simulating heat).

The variance components of the genetic main effects with GB and l were low (0.064 and 0.061 for MDsl-GB and MDel-GB, respectively), indicating low exchange of information between environments. The most influential variance components were related to the G×E, $\sigma_{gE}^{2}$. For example, for models MDs-GB, the variance component $\sigma_{gE}^{2}$ is 0.618 and 0.636 for MDsl-GB, whereas it increases to $\sigma_{gE}^{2}$ = 1.482 for MDs-GK and to $\sigma_{gE}^{2}$ = 1.49 for MDsl-GK (Table 8); this result indicates the importance of G×E interaction. The influence of the random component l in this data set is negligible. The variance components related to specific environments are similar for the five environments and for MDe models with and without random component l.

**Figure 4:**
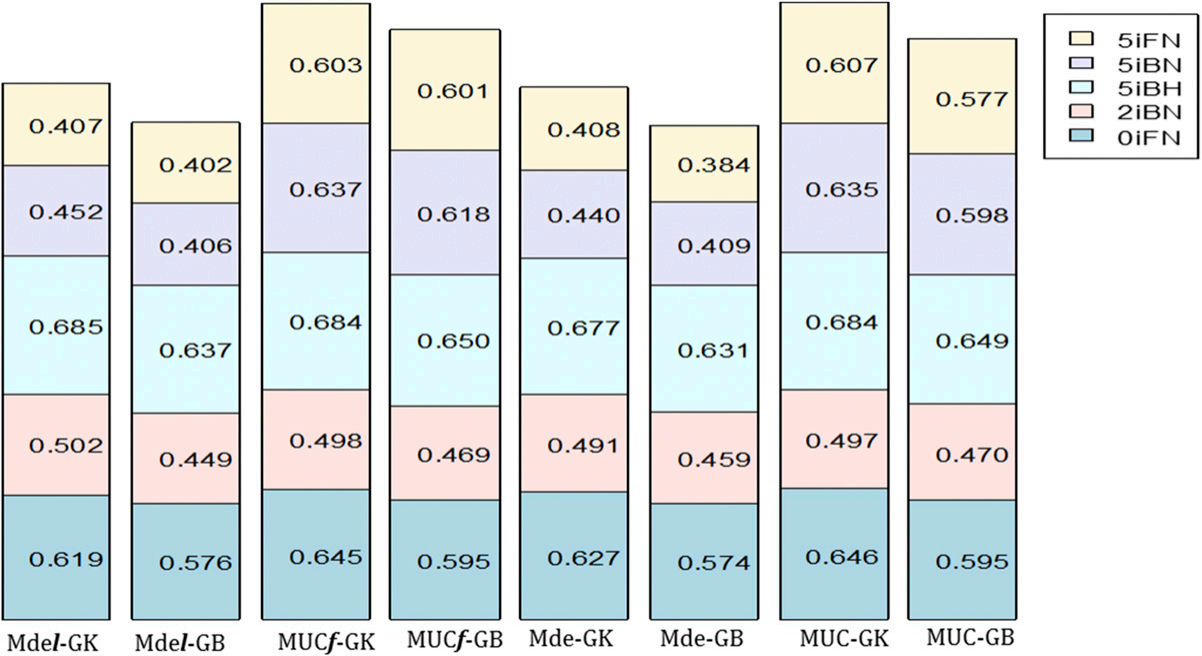
Plot of the prediction accuracy using Pearson’s correlation for each of the 5 environments (0iFN, 2iBH, 5iBH, 5iBN, 5iFN) of wheat data set WHE5 for the proposed models MDe***l***-GK, MDe***l***-GB, MUC***f***-GK, MUC***f***-GB, MDe-GK, MDe-GB, MUC-GK, and MUC-GB.

Among the 12 model-method combinations, the best predictive models were MDel-GK and MDe-GK in all locations (Table 9, Figure 4). However, models MDsl-GK and MDs-GK also had relatively high prediction accuracies that were very similar to those of models MDel-GK and MDe-GK. Similar results were found for models with linear kernel GB (Table 9). Models with the random intercept l showed no increase in prediction accuracy (values of $\sigma_{l}^{2}$ close to zero) as compared to models without l.

**Table 9:** WHEAT WHE5 data set. Mean Pearson’s correlation (50 partitions) of each environment for random cross-validation CV2, for the multi-environment models, main genotypic effect model (MM), single variance G×E deviation model (MDs), environment-specific variance G×E deviation model (MDe), multi-environment unstructured covariance models (MUC and MUCf) with two kernels, GBLUP (GB) and Gaussian kernel (GK) for grain yield with the proposed random effect l and without the random effect l (standard deviation in parentheses)

	Proposed models with random effects l and ***f***							
Environment*	MMl-GK	MMl-GB	MDsl-GK	MDsl-GB	Mdel**-**GK	MDel**-**GB	MUC***f***-GK MUC***f***-GB	
0iFN	0.309 (0.05)	0.301 (0.05)	0.610 (0.04)	0.58 (0.03)	0.619 (0.04)	0.576 (0.04)	0.645 (0.05)	0.595 (0.05)
2iBN	0.186 (0.06)	0.191 (0.05)	0.495 (0.04)	0.453 (0.03)	0.502 (0.04)	0.449 (0.04)	0.498 (0.06)	0.469 (0.06)
5iBH	0.23 (0.06)	0.267 (0.05)	0.678 (0.02)	0.631 (0.04)	0.685 (0.03)	0.637 (0.03)	0.684 (0.03)	0.650 (0.04)
5iBN	0.262 (0.05)	0.256 (0.05)	0.456 (0.04)	0.430 (0.04)	0.452 (0.04)	0.406 (0.05)	0.637 (0.04)	0.618 (0.04)
5iFN	0.266 (0.05)	0.247 (0.05)	0.418 (0.05)	0.401 (0.05)	0.407 (0.05)	0.402 (0.04)	0.603 (0.05)	0.601 (0.05)
	Proposed models without random effects l and ***f***							
Environment	MM-GK	MM-GB	MDs-GK	MDs-GB	MDe-GK	MDe**-**GB	MUC-GK MUC-GB
0iFN	0.321 (0.05)	0.303 (0.05)	0.621 (0.03)	0.572 (0.03)	0.627 (0.04)	0.574 (0.03)	0.646 (0.05)	0.595 (0.05)
2iBN	0.215 (0.04)	0.211 (0.05)	0.49 (0.05)	0.451 (0.04)	0.491 (0.05)	0.459 (0.04)	0.497 (0.06)	0.470 (0.06)
5iBH	0.248 (0.06)	0.284 (0.05)	0.675 (0.02)	0.646 (0.03)	0.677 (0.03)	0.631 (0.03)	0.684 (0.03)	0.649 (0.04)
5iBN	0.255 (0.04)	0.245 (0.05)	0.452 (0.04)	0.407 (0.04)	0.440 (0.05)	0.409 (0.05)	0.635 (0.04)	0.598 (0.04)
5iFN	0.251 (0.05)	0.245 (0.05)	0.405 (0.04)	0.394 (0.04)	0.408 (0.04)	0.384 (0.04)	0.607 (0.05)	0.577 (0.05)

* Environments are described by a sequence of codes: 0i, 2i and 5i denote the number of irrigation cycles; B/F denotes whether the planting system was ‘bed’ (B) or ‘flat’ (F); N/H denotes whether planting date was normal (N) or late (H, simulating heat).

The comparison of the prediction accuracy of these 12 model-method combinations with the mean prediction accuracy of models MUC***f*** and MUC (Figure 4) indicated the higher mean prediction accuracy of MUC***f*** and MUC over the mean prediction accuracy of the proposed models with (or without) the random effect l. For this data set, the prediction accuracies of CV1 were similar to those found under CV2 (Table S4, Appendix 2).

In summary, the complex G×E interaction in this data set is expressed by the large variance component $\sigma_{ge}^{2}$. Models with random component l did not increase the prediction accuracy of the corresponding models without l (reflected in their values of $\sigma_{l}^{2}$ close to zero). Of the 12 model-method combinations, models MDel-GK and MDe-GK gave the highest prediction accuracies. However, the best predictive models overall and for each environment were MU***f*** and MUC.

## Discussion

### Effect of random component l

From a statistical perspective, the mixed models can better explain the variation among lines in environments (G×E) by considering two factors: environments and lines. The environmental effects ($\beta_{E}$) are considered as fixed effects with the relationship$\;Z_{E}\beta_{E}$; however, the effects of the lines are considered random in $Z_{g}g+Z_{g}l$ for model MMl. The g is the common random effect of each line derived from the markers and l is considered the random intercept for each line. If we make the transformation $g^{*}=Z_{g}g$, as in López-Cruz *et al.* (2015), then $g^{*}∼N($**0**, $\sigma_{g}^{2}Z_{g}KZ_{g}^{\prime }),$ where matrix $Z_{g}KZ_{g}^{\prime }$ comprises submatrices (or blocks) where the submatrices off the block diagonal generated the exchange of information between environments with positive correlations. As discussed by López-Cruz *et al.* (2015), this exchange of information is not effective when there are negative correlations between sites (or environments) due to the fact that they are based on $\sigma_{g}^{2}$. Similarly, if $l^{*}=Z_{g}l$, then **$l^{*}∼N($**0, $\sigma_{l}^{2}Z_{g}IZ_{g}^{\prime })$ and the exchange of information occurred in the submatrices off the block diagonal between the environments with positive correlations and when $\sigma_{l}^{2}$ is not zero. On the other hand, in models MDsl and MDel, the component l has influence only when there is exchange of information across environments and the G×E is simple; otherwise, as in the WHE5 data set, the contribution of l is negligible when the G×E is complex.

The random effects ***l*** are independent and identically distributed (iid) thus do not have the possibility of exchanging of information from tested lines to untested lines and therefore do not have any estimate of these values if no evaluation data on a line exists (CV1). Then, when trying to predict values of untested lines, only available information between lines come from the ***g*** part of the model. In a number of cases, substantial variation for the ***l*** effects were found suggesting that the additive part of the model (***g***) is not capturing the total genetic value very well. In these cases, since usually the GK method did as well as the GB with ***l*** model, there is a major advantage to the GK method in that it can better predict untested genotypes since the marker information is being used in a way that captures more of the genetic variation. On the other hand, if the breeder is concerned about gain from selection following intermating and generating a new population, the breeder should only be selecting based on the additive breeding values and realizing that the breeding values are not the complete genotypic value (commercial value), such that response to selection after intermating will be less than expected based on total genetic variance.

### Effects of including G×E interaction

In general, results show that when GBLUP is used for prediction under random cross-validation CV2, models MDsl-GB and MDel-GB that incorporate G×E had higher prediction accuracy than models MDs-GB and MDe-GB also with G×E. This improvement depends on $\sigma_{l}^{2}$ and the magnitude of the correlations between environments. For maize data sets (HEL and USP) with positive sample correlations between environments, models MDsl**-**GB and MDel**-**GB had higher prediction accuracy than models MDs-GB and MDe-GB, whereas in wheat data set WHE1, models MDsl and MDel had better prediction accuracy than models MDs and MDe only in environments with positive correlations. Finally, for environments in wheat data set WHE5 with negligible $\sigma_{l}^{2}$, the accuracy of models MDsl-GB and MDel-GB did not improve much over that of models MDs-GB and MDe-GB without l.

### Effects of including the Gaussian kernel

In general, models MDs and MDe with the Gaussian kernel (GK) had higher prediction accuracy than models with GB, although these differences were smaller for models MDsl and MDel. When GK models were better than GB models, results show that $\sigma_{l}^{2}$ was negligible for GK models and when the prediction accuracy of MDsl and MDel was only slightly superior to that of models MDs and MDe (as in maize data set HEL). On the contrary, when using GK, the prediction accuracy was not better than when using GB, as in the case of maize data set USP; then the contribution of $\sigma_{l}^{2}$ was important and the prediction accuracy of MDsl and MDel was superior to that of their counterparts MDs and MDe. These results indicate that models with random intercepts are useful when used with the linear kernel (GB) but not when used with the Gaussian kernel (GK). This is because the GK method without l explains most of the genetic variance (additive and epistasis effects) between lines with negligible genetic residuals that are not picked up by the ***l***.

### The effect of the sample covariance among environments

The behavior of the covariance between observations of the *i*th line in the *j*th and *j*’th environments explains some of the results obtained in the four data sets. The covariance between $y_{ij}$ and $y_{ij^{’}}$ of models MM, MDs and MDe is the same; it is determined by the genetic variance component $\sigma_{g}^{2}$. It would be expected that the estimate of $\sigma_{g}^{2}$ would be proportional to the sample covariance of the observations. This only occurred when the sample covariances were positive because $\sigma_{g}^{2}$ can take only positive values; when the sample covariances between some environments are negative, this distorts the estimations of the genetic variance component ($\sigma_{g}^{2}$) and therefore affects the prediction accuracy of the unobserved phenotypes of the lines in the testing set.

On the other hand, when the sample covariance between $y_{ij}$ and $y_{ij^{’}}$ of models MMl, MDsl and MDel is determined by the summation $\sigma_{g}^{2}$ + $\sigma_{l}^{2}$, then the higher $\sigma_{l}^{2}$, the higher the estimated sample covariance (association) of the lines in environments and, therefore, the higher the prediction accuracy compared with those achieved by models MM, MDs and MDe (without the random effect l). Again, the presence of negative sample covariances distorts the behavior of the estimated genetic variance components and this negatively affects the prediction accuracy of these models.

### Models With G×E With the Kronecker product *vs.* models With G×E With the Hadamard product

Less restrictive G×E genomic-enabled prediction models that allow any covariance value between environments had better prediction accuracy than models with more restrictive assumptions at the level of association between lines in environments affecting the estimation of the genetic variance components. Less restrictive models consider variance-covariance matrices represented by the Kronecker product of the variances and covariances of the environmental and genetic values (with the linear or non-linear kernels constructed with the markers) (Burgueño *et al.* 2012; Cuevas *et al.* 2017). When a random intercept (f) is added to these models based on the Kronecker product (Cuevas *et al.* 2017), the genomic-enabled prediction accuracy increased for random cross-validation CV2 and for environments with negative sample covariance. These advantages of the G×E genomic-enabled prediction models using the Kronecker product for defining variance-covariance environmental matrices with negative or zero environmental relationship over the Hadamard product defined by models MDsl and MDel are less when sample covariances between environments are all positive. The disadvantages of models with Kronecker products are that defining and measuring environmental stability is not clear, plus they demand higher computing resources compared to G×E genomic-enabled prediction models using the Hadamard product.

### Required computing time for fitting the models

We performed all the analyses in an Ubuntu Linux server with 256 GB of RAM and 32 CPUs core. To compare the computing time, we counted the mean computing time in seconds for fitting one random partition for random cross-validation CV1 for the maize data set HELIX with the same number of 50 partitions and the same number of iterations in the model. For the models with G×E without l or ***f***, the mean computing time for one random partition was 290, 319, and 3110 for models MDs, MDe, and MUC, respectively. For models with G×E with random intercept l or ***f***, the mean computing time for one random partition was 489, 541, and 4938 for models MDsl, MDel, and MUC***f***, respectively. The differences in computing time between models MDs and MDe are low, but for model MUC, the required mean computing time needed to fit the model increased 10 times for one random partition.

### Advantages and disadvantages of the proposed models

In general, G×E genomic-enabled prediction models MDsl and MDel had similar prediction accuracy and, in both cases, environmental stability and G×E can be assessed and measured. Furthermore, in models MDsl and MDel, when the sample correlation among environments is positive, their prediction accuracy is similar or slightly higher than the accuracy achieved with the more flexible Kronecker product models (Burgueño *et al.* 2012; Cuevas *et al.* 2017) for the variance-covariance matrices. The advantage of models MDsl and MDel with the Hadamard product for the variance-covariance is that they can perform highly dimensional matrix operations very fast and, therefore, save time when fitting these models. The BGGE software developed by Granato *et al.* (2017) is indeed an example of this efficiency for fitting models MDsl and MDel by means of the Hadamard product.

When the main objective is prediction accuracy, we recommend checking for sample covariance (or correlations) between environments before using MDsl and MDel G×E genomic-enabled prediction models. Models MDsl, MDel, MDs and MDe are recommended when the sample correlations are positive and not close to zero. We also recommend fitting models MDsl, and MDel to the training set and estimating the variance component of the random intercept $\sigma_{l}^{2}$; if it is negligible, only models MDs and MDe should be used. When the number of lines in each environment is not the same, models MDsl, MDel, MDs, and MDe can be efficiently fitted with the BGGE software, whereas models MUC***f*** and MUC of Cuevas *et al.* (2017) with an unbalanced number of lines in each environments require intensive computational resources.

### CONCLUSIONS

Results indicate that when the sample phenotypic correlations between environments were intermediate to moderate (HEL, USP), models with G×E with random intercept l (MDsl, MDel) and Gaussian kernel (GK) had the advantages of other models without their disadvantages. These models allow: (i) finding regions of the chromosomes with environmental stability (Jarquín *et al.* 2014; López-Cruz *et al.* 2015), (ii) the fitted computing time is fast (Granato *et al.* 2017), and (iii) increasing the prediction accuracy in the CV2 to a level of the Gaussian kernels of Cuevas *et al.* (2016) and Sousa *et al.* (2017) or other more flexible models such as those used by Burgueño *et al.* (2012) and Cuevas *et al.* (2017). For sample low or negative phenotypic correlations like in data sets WHE1, WHE5, the prediction accuracy of model MUC***f*** with GK of Cuevas *et al.* (2017) is the one that should be used.

Including the random intercept l for each line made it possible to capture some extra genetic variability. Models MDs and MDe assessed the complexity of the genomic G×E present in the two maize data sets (with all environments with positive correlations) by means of the Hadamard product between markers and environments as in models from Jarquín *et al.* (2014) (MM, and MDs) and López-Cruz *et al.* (2015) (MDe). For the two maize data sets with positive sample correlations among environments, the Hadamard models MM, MDs and MDe with l had similar prediction accuracies as models MUC***f*** and MUC that use a Kronecker product for assessing G×E. The advantage of models MMl, MDsl, and MDel over models MUC***f*** and MUC is shorter computing time when the number of lines in different environments is very unbalanced, as in the case of the two maize data sets.

For the two wheat data sets, the number of lines in each environment is the same. However, in view of the fact that the sample correlation among environments is not positive for all pair-wise environment combinations, using models MM, MDs and MDe with or without l is less favorable than using models MUC***f*** and MUCwith a Kronecker product for modeling G×E. The reduced prediction accuracy of the Hadamard product models *vs.* the Kronecker product models indicated the flexibility of models MUC***f*** and MUC for assessing complex G×E multi-environment data sets. Regardless of: (i) whether l is included or not, and (ii) the type of data set at hand (with more or less complex G×E) and the balanced or unbalanced data structure, the prediction accuracy of the Gaussian kernel was better than the prediction accuracy of the linear kernel GBLUP for all four data sets.

## APPENDIX 1

### The multi-environment main genotypic effect model (MM)

The multi-environment model (MM) considers the fixed effects of environments ($\beta_{E}$), as well as the random genetic effects across environments (g)

$$y=\mu 1+Z_{E}\beta_{E}+Z_{g}g+\varepsilon$$ (1)

where $y={\left(y_{1},\ldots ,y_{j},\ldots y_{m}\right)}^{\prime }\;$is a vector with the observations $y_{j}$ of the *j*^th^ environment (j=1,…,m), each of size $n_{j}$, such that one line in one environment represents the $y_{ij}$ observation of the i^th^ line $\left(i=1,\ldots ,\;n_{j}\right)$ in the *j*^th^ environment. The scalar μ is a general mean and the vector 1 is of size $\overset{m}{\underset{j=1}{\sum }}n_{j}\times 1.$ The fixed effects of the environment for the data used in this study are modeled with the incidence matrix of the environments $Z_{E}$ of order $\overset{m}{\underset{j=1}{\sum }}n_{j}\times m$, where the parameters to be estimated are the intercept for each environment ($\beta_{E}$) with the vector $\beta_{E}$ of order m×1. Incorporating other fixed effects into the model is straightforward.

The random vector of genetic effects g  follows a multivariate normal distribution with mean zero and a covariance matrix K, that is, $g∼N(0,\sigma_{g}^{2}K)$, where the vector g  of order n×1 represents the genetic random effects across all environments for each line, and the kernel matrix K is a symmetric semidefinite positive matrix constructed with molecular markers of order n×n. If the number of lines is the same in each environment, then $n=n_{1}=\ldots =n_{j}=\ldots =n_{m}$; otherwise, when there are different numbers of lines in each environment, n represents the number of unique lines included in the model in some environments. The incidence matrix $Z_{g}$ connects genotypes with phenotypes for each environment, with order $\overset{m}{\underset{j=1}{\sum }}n_{j}\times n$. Variance component $\sigma_{g}^{2}$ is the genetic variance of the lines across all environments and represents the sensitivity or environmental stability. Finally, the random errors are assumed to be homoscedastic and independent, $\varepsilon ∼N(0,\sigma^{2}I)$, where $\sigma^{2}$ is the error variance.

### The multi-environment single variance genotype × environment interaction deviation model (MDs)

This model adds to the MM model the random interaction effects of the environments with the genetic information of the lines ($ge_{ij}$) (Sousa *et al.*, 2017; Jarquín *et al.*, 2014):

$$y=\mu 1+Z_{E}\beta_{E}+Z_{g}g+\mathrm{ge}+\varepsilon$$ (2)

The vector of random effects G×E interaction, ge, has a multivariate normal distribution, $\mathrm{ge}∼N(0,\;\left[Z_{g}KZ_{g}^{\prime }\right]\circ \left[Z_{E}Z_{E}^{\prime }\right]\sigma_{ge}^{2}$), where (∘) is the Hadamard product operator, and $\sigma_{ge}^{2}$ is the variance component of the G×E interaction. Matrix $\left[Z_{g}KZ_{g}^{\prime }\right]\circ \left[Z_{E}Z_{E}^{\prime }\right]\;$is a block diagonal constructed with the matrices K **($K_{1}\ldots K_{j}$**…$K_{m})\;$for each environment; therefore, there is no exchange (borrowing) of information between environments:

$$\left[Z_{g}KZ_{g}^{\prime }\right]\circ \left[Z_{E}Z_{E}^{\prime }\right]=\left[\begin{array}{ccccc} K_{1} & \cdots & 0 & \cdots & 0\\ \vdots & \ddots & \vdots & \ddots & \vdots \\ 0 & \cdots & K_{j} & \cdots & 0\\ \vdots & \ddots & \vdots & \ddots & \vdots \\ 0 & \cdots & 0 & \cdots & K_{m} \end{array}\right]$$

### Multi-environment environment-specific variance genotype × environment deviation model (MDe)

The multi-environment, environment-specific variance genotype × environment deviation model (MDe) (López-Cruz *et al.*, 2015) differs from MDs in how the random interaction component is modeled:

$$y=\mu 1+Z_{E}\beta_{E}+Z_{g}g+g_{E}+\varepsilon$$ (3)

where g is the main genetic effect across all the environments and $g_{E}$ represents the specific genetic effects in each environment such that $g_{E}∼N(0,K_{E})$, where $K_{E}$ is a matrix block diagonal generated with individuals included in each environment:

$$K_{E}=\left[\begin{array}{ccccc} \sigma_{g_{E_{1}}}^{2}K_{1} & \cdots & 0 & \cdots & 0\\ \vdots & \ddots & \vdots & \ddots & \vdots \\ 0 & \cdots & \sigma_{g_{E_{j}}}^{2}K_{j} & \cdots & 0\\ \vdots & \ddots & \vdots & \ddots & \vdots \\ 0 & \cdots & 0 & \cdots & \sigma_{g_{E_{m}}}^{2}K_{m} \end{array}\right]$$

with a variance component specific for each environment $\sigma_{u_{E_{j}}}^{2}$(Sousa *et al.*, 2017).

## APPENDIX 2

**Table S1.** Maize HEL data set. Mean Pearson’s correlation (50 partitions) of each location for random cross-validation CV1, for the multi-environment models, main genotypic effect model (MM), single variance G×E deviation model (MDs), environment-specific variance G×E deviation model (MDe), multi-environment unstructured covariance models (MUC and MUCf) with two kernels, GBLUP (GB) and Gaussian kernel (GK) for grain yield with the proposed random effect l and without the random effect l (standard deviation in parentheses)

	Proposed models with random effects l and ***f***							
Location*	MMl-GK	MMl-GB	MDsl-GK	MDsl-GB	MDel**-**GK	MDel**-**GB	MUC***f***-GK MUC***f***-GB	
IP	0.571 (0.1)	0.439 (0.12)	0.745 (0.05)	0.644 (0.1)	0.749 (0.06)	0.634 (0.09)	0.756 (0.06)	0.659 (0.06)
NM	0.503 (0.08)	0.354 (0.09)	0.532 (0.08)	0.385 (0.11)	0.525 (0.09)	0.365 (0.11)	0.537 (0.08)	0.381 (0.11)
PM	0.661 (0.06)	0.574 (0.07)	0.753 (0.05)	0.682 (0.07)	0.753 (0.04)	0.685 (0.05)	0.751 (0.04)	0.685 (0.05)
SE	0.347 (0.09)	0.202 (0.11)	0.505 (0.08)	0.370 (0.1)	0.513 (0.08)	0.366 (0.09)	0.489 (0.09)	0.349 (0.09)
SO	0.442 (0.1)	0.287 (0.09)	0.552 (0.08)	0.402 (0.1)	0.552 (0.08)	0.395 (0.12)	0.551 (0.08)	0.39 (0.09)
	Proposed models without random effects l and ***f***							
Location*	MMl-GK	MM-GB	MDs-GK	MDs-GB	MDe-GK	MDe**-**GB	MUC-GK MUC-GB
IP	0.575 (0.09)	0.426 (0.11)	0.752 (0.06)	0.607 (0.08)	0.755 (0.05)	0.618 (0.09)	0.758 (0.05)	0.641 (0.08)
NM	0.506 (0.09)	0.361 (0.07)	0.54 (0.09)	0.394 (0.08)	0.538 (0.09)	0.394 (0.08)	0.545 (0.06)	0.391 (0.1)
PM	0.662 (0.06)	0.533 (0.07)	0.758 (0.05)	0.662 (0.07)	0.754 (0.05)	0.671 (0.04)	0.754 (0.04)	0.669 (0.05)
SE	0.346 (0.1)	0.219 (0.1)	0.527 (0.06)	0.321 (0.1)	0.524 (0.08)	0.339 (0.09)	0.505 (0.07)	0.319 (0.11)
SO	0.455 (0.1)	0.293 (0.11)	0.576 (0.07)	0.376 (0.1)	0.555 (0.09)	0.383 (0.11)	0.56 (0.07)	0.377 (0.1)

* Locations are: IP: Ipiaçú-MG, NM: Nova Mutum-MT, PM: Pato de Minas-MG, SE: Sertanópolis-PR, and SO: Sorriso-MT.

**Table S2.** Maize USP data set. Mean Pearson’s correlation (50 partitions) of each location for random cross-validation CV1, for the multi-environment models, main genotypic effect model (MM), single variance G×E deviation model (MDs), environment-specific variance G×E deviation model (MDe), multi-environment unstructured covariance models (MUC and MUCf) with two kernels, GBLUP (GB) and Gaussian kernel (GK) for grain yield with the proposed random effect l and without the random effect l (standard deviation in parentheses)

	Proposed models with random effects l and ***f***							
Environment*	MMl-GK	MMl-GB	MDsl-GK	MDsl-GB	MDel**-**GK	MDel**-**GB	MUC***f***-GK MUC***f***-GB	
P-LN	0.28 (0.06)	0.272 (0.07)	0.307 (0.06)	0.293 (0.07)	0.303 (0.06)	0.286 (0.07)	0.294 (0.07)	0.286 (0.06)
P-IN	0.304 (0.06)	0.298 (0.08)	0.335 (0.08)	0.329 (0.06)	0.335 (0.07)	0.332 (0.08)	0.331 (0.08)	0.327 (0.06)
A-LN	0.287 (0.07)	0.283 (0.05)	0.305 (0.08)	0.31 (0.06)	0.303 (0.06)	0.309 (0.06)	0.321 (0.08)	0.309 (0.07)
A-IN	0.389 (0.07)	0.386 (0.08)	0.42 (0.07)	0.413 (0.07)	0.425 (0.07)	0.422 (0.06)	0.418 (0.05)	0.417 (0.07)
	Proposed models without random effects l and ***f***							
Environment*	MMl-GK	MM-GB	MDs-GK	MDs-GB	MDe-GK	MDe**-**GB	MUC-GK MUC-GB
P-LN	0.286 (0.07)	0.278 (0.05)	0.305 (0.05)	0.289 (0.07)	0.313 (0.08)	0.295 (0.07)	0.311 (0.06)	0.30 (0.06)
P-IN	0.285 (0.08)	0.313 (0.06)	0.324 (0.06)	0.332 (0.07)	0.324 (0.07)	0.33 (0.05)	0.318 (0.05)	0.341 (0.06)
A-LN	0.262 (0.07)	0.292 (0.07)	0.278 (0.06)	0.313 (0.06)	0.285 (0.07)	0.308 (0.06)	0.300 (0.06)	0.318 (0.07)
A-IN	0.365 (0.06)	0.391 (0.07)	0.395 (0.06)	0.415 (0.07)	0.403 (0.07)	0.417 (0.06)	0.406 (0.05)	0.424 (0.07)

* Environments are: Anhumas ideal N (A-IN), Anhumas low N (A-LN), Piracicaba ideal N (P-IN) and Piracicaba low N (P-LN)

**Table S3.** Wheat data set WHE1. Mean Pearson’s correlation (50 partitions) of each location for random cross-validation CV1, for the multi-environment models, main genotypic effect model (MM), single variance G×E deviation model (MDs), environment-specific variance G×E deviation model (MDe), multi-environment unstructured covariance models (MUC and MUCf) with two kernels, GBLUP (GB) and Gaussian kernel (GK) for grain yield with the proposed random effect l and without the random effect l (standard deviation in parentheses)

	Proposed models with random effects l and ***f***							
Environment	MMl-GK	MMl-GB	MDsl-GK	MDsl-GB	MDel**-**GK	MDel**-**GB	MUC***f***-GK MUC***f***-GB	
E1	0.048 (0.06)	0.054 (0.07)	0.545 (0.05)	0.512 (0.05)	0.558 (0.05)	0.510 (0.05)	0.560 (0.05)	0.515 (0.06)
E2	0.397 (0.06)	0.405 (0.06)	0.49 (0.06)	0.476 (0.06)	0.48 (0.05)	0.474 (0.05)	0.472 (0.05)	0.478 (0.05)
E3	0.368 (0.07)	0.373 (0.06)	0.405 (0.05)	0.366 (0.06)	0.416 (0.06)	0.399 (0.05)	0.413 (0.06)	0.386 (0.06)
E4	0.341 (0.06)	0.329 (0.05)	0.472 (0.04)	0.439 (0.05)	0.467 (0.05)	0.441 (0.06)	0.464 (0.06)	0.450 (0.04)
	Proposed models without random effects l and ***f***							
Environment	MMl-GK	MM-GB	MDs-GK	MDs-GB	MDe-GK	MDe**-**GB	MUC-GK MUC-GB
E1	0.066 (0.06)	0.049 (0.06)	0.544 (0.05)	0.472 (0.06)	0.539 (0.04)	0.495 (0.05)	0.571 (0.04)	0.513 (0.04)
E2	0.416 (0.06)	0.414 (0.06)	0.476 (0.05)	0.475 (0.06)	0.472 (0.05)	0.464 (0.05)	0.465 (0.05)	0.454 (0.05)
E3	0.377 (0.05)	0.384 (0.05)	0.397 (0.05)	0.388 (0.06)	0.423 (0.05)	0.392 (0.05)	0.405 (0.05)	0.381 (0.05)
E4	0.339 (0.05)	0.339 (0.05)	0.469 (0.04)	0.437 (0.04)	0.46 (0.05)	0.416 (0.05)	0.456 (0.05)	0.418 (0.05)

**Table S4.** Wheat data set WHE5. Mean Pearson’s correlation (50 partitions) of each location for random cross-validation CV1, for the multi-environment models, main genotypic effect model (MM), single variance G×E deviation model (MDs), environment-specific variance G×E deviation model (MDe), multi-environment unstructured covariance models (MUC and MUCf) with two kernels, GBLUP (GB) and Gaussian kernel (GK) for grain yield with the proposed random effect l and without the random effect l (standard deviation in parentheses)

	Proposed models with random effects l and ***f***							
Environment*	MMl-GK	MMl-GB	MDsl-GK	MDsl-GB	MDel**-**GK	MDel**-**GB	MUC***f***-GK MUC***f***-GB	
0iFN	0.348 (0.05)	0.301 (0.05)	0.601 (0.04)	0.553 (0.03)	0.611 (0.04)	0.555 (0.04)	0.614 (0.03)	0.554 (0.03)
2iBN	0.217 (0.05)	0.201 (0.05)	0.474 (0.04)	0.431 (0.04)	0.47 (0.05)	0.439 (0.04)	0.475 (0.04)	0.448 (0.04)
5iBH	0.321 (0.05)	0.35 (0.05)	0.67 (0.03)	0.635 (0.03)	0.668 (0.03)	0.634 (0.03)	0.679 (0.03)	0.633 (0.03)
5iBN	0.163 (0.06)	0.136 (0.06)	0.399 (0.04)	0.353 (0.05)	0.395 (0.05)	0.345 (0.06)	0.401 (0.04)	0.358 (0.05)
5iFN	0.084 (0.06)	0.082 (0.06)	0.334 (0.04)	0.309 (0.04)	0.328 (0.05)	0.306 (0.05)	0.336 (0.05)	0.315 (0.04)
	Proposed models without random effects l and ***f***							
Environment*	MMl-GK	MM-GB	MDs-GK	MDs-GB	MDe-GK	MDe**-**GB	MUC-GK MUC-GB
0iFN	0.341 (0.05)	0.288 (0.05)	0.61 (0.04)	0.562 (0.03)	0.612 (0.03)	0.557 (0.03)	0.625 (0.04)	0.558 (0.04)
2iBN	0.205 (0.05)	0.216 (0.05)	0.478 (0.05)	0.439 (0.05)	0.473 (0.05)	0.436 (0.04)	0.476 (0.05)	0.429 (0.06)
5iBH	0.323 (0.04)	0.333 (0.05)	0.67 (0.02)	0.624 (0.03)	0.662 (0.03)	0.627 (0.03)	0.680 (0.03)	0.638 (0.03)
5iBN	0.171 (0.05)	0.163 (0.05)	0.397 (0.05)	0.357 (0.04)	0.405 (0.04)	0.356 (0.04)	0.407 (0.04)	0.354 (0.05)
5iFN	0.107 (0.05)	0.114 (0.06)	0.33 (0.05)	0.311 (0.04)	0.329 (0.05)	0.307 (0.05)	0.337 (0.04)	0.303 (0.04)

* Environments are described by a sequence of codes: 0i, 2i and 5i denote the number of irrigation; B/F denotes whether the planting system was ‘bed’ (B) or ‘flat’ (F); N/H denotes whether planting date was normal (N) or late (H, simulating heat).

## APPENDIX 3

**Table A1.** Table A1. Phenotypic Pearson’s correlations among locations for grain yield for the four data sets HEL (maize), USP (maize), WHE1 (wheat), WHE2 (wheat). For HEL and USP maize data sets, the number in parentheses below each location’s name indicates the number of lines sown. For the two data sets in the wheat experiments (WHE1 and WHE2), the number of wheat lines is given in parentheses

	HEL (452 maize lines) (Sousa *et al.* 2017)							
Location*	Ipiaçú (IP) (247)	Nova Mutum (NM) (330)		Pato de Minas (PM) (452)		Sertanópolis (SE) (367)		Sorriso (SO) (330)
Nova Mutum (NM)	0.46	—		—		—		—
Pato de Minas (PM)	0.51	0.44		—		—		—
Sertanópolis (SE)	0.29	0.36		0.30		—		—
Sorriso (SO)	0.43	0.48		0.39		0.38		—
	USP (739 maize lines) (Sousa *et al.* 2017)							
Environment	Piracicaba-LN (P-LN) (731)		Piracicaba-IN (P-IN) (732)		Anhumas-LN (A-LN) (731)		Anhumas-IN (L-IN) (737)	
Piracicaba-IN (P-LN)	0.54		—		—		—	
Anhumas-LN (P-IN)	0.31		0.35		—		—	
Anhumas-IN (A-IN)	0.43		0.47		0.47		—	
	WHE1 (599 wheat lines)							
Location*	E1	E2		E3		E4		
E2	−0.19	—		—		—		
E3	−0.19	0.661		—		—		
E4	−0.12	0.411		0.388		—		
	WHE5 (807 wheat lines)							
Location*	0iFN	2iBN		5iBH		5iBN		5iFN
2iBN	0.166	—		—		—		—
5iBH	0.30	−0.033		—		—		—
5iBN	−0.10	0.122		−0.091		—		—
5iFN	−0.01	0.035		0.023		0.546		—

* Locations in HEL data set are: IP: Ipiaçú-MG, NM: Nova Mutum-MT, PM: Pato de Minas-MG, SE: Sertanópolis-PR, and SO: Sorriso-MT. Locations in USP data set are: IN = ideal Nitrogen; LN = low nitrogen. In WHE5 data set, environments are described by a sequence of codes: 0i, 2i and 5i denote the number of irrigations; B/F denotes whether the planting system was ‘bed’ (B) or ‘flat’ (F); N/H denotes whether planting date was normal (N) or late (H, simulating heat).
